# Development and Initial Assessment of a Novel and Customized Bile Duct Simulator for Handsewn Anastomosis Training

**DOI:** 10.7759/cureus.31749

**Published:** 2022-11-21

**Authors:** Julia Micallef, Mithusa Sivanathan, Krystina M Clarke, Merieme Habti, Florence Bénard, Léamarie Meloche-Dumas, Erica Patocskai, Adam Dubrowski

**Affiliations:** 1 Health Sciences, Ontario Tech University, Oshawa, CAN; 2 Medical Pedagogy, University of Montreal Health Centre, Montreal, CAN; 3 General Surgery, University of Montreal Health Centre, Montreal, CAN; 4 Surgical Oncology, University of Montreal Health Centre, Montreal, CAN

**Keywords:** surgical skills-based training, additive manufacturing, three-dimensional printing, simulation-based medical education, bile duct anastomosis

## Abstract

Simulation-based medical education allows for the training and maintenance of healthcare skills in a safe and controlled environment. In this technical report, the development and initial evaluation of a bile duct anastomosis simulator are described. The simulator was developed using additive manufacturing techniques such as three-dimensional (3D) printing and silicone work. The final product was produced by maxSIMhealth, a research lab at Ontario Tech University (Oshawa, ON, Canada), and included four individual silicone bile ducts, based on the expert opinions from surgeons at the Centre Hospitalier de l'Université de Montréal (Montreal, QC, Canada), and a 3D-printed maxSIMclamp, which was described in a previous report. The evaluation was conducted by nine individuals consisting of surgeons, surgical residents, and medical students to assess the fidelity, functionality, and teaching quality of the simulator. The results from the evaluation indicate that the simulator needs to improve its fidelity by being softer, thinner, and beige. On the other hand, the results also indicate that this simulator is extremely durable and can be used as a training tool for surgical residents with some minor improvements.

## Introduction

Simulation-based medical education (SBME) allows healthcare professionals, educators, and learners to practice clinical procedures in a safe and controlled environment through the use of simulators [1]. While using simulators reduces patient harm and avoids ethical issues found with animal models, commercially available simulators can often be expensive and not customizable to the learner’s needs [1].

In the past, we have provided an alternative way to produce simulators that are anatomically accurate, customizable, and cost-effective through the implementation of additive manufacturing (AM) technologies, such as three-dimensional (3D) printing and silicone work [2-4]. 3D printing has been introduced into SBME as a low-cost alternative to costly simulators for learning procedural skills [5]. The procedural skill we are interested in creating a simulator for is bile duct anastomosis (BDA). A BDA usually involves one of two biliary reconstruction techniques: either an end-to-end ductal anastomosis or a Roux-en-Y hepaticojejunostomy. The former is often used in liver transplantation, while the latter is commonly seen when repairing iatrogenic bile duct injuries or during a Whipple procedure [6].

This technical report has two purposes: (1) to describe the development of a BDA simulator using a collaborative approach with AM techniques and (2) to provide an initial evaluation of the BDA simulator.

## Technical report

This report is described using a modified context, input, process, and product (CIPP) simulator to optimize applicability to various learning environments, educational contexts, inputs, processes, and expected outcomes [7].

Context

The BDA simulator was designed to train general surgery residents from the University of Montreal (Montreal, QC, Canada) in this skill. It was co-designed by two collaborative groups: graduate students from maxSIMhealth (https://maxsimhealth.com/), a research laboratory located at Ontario Tech University (Oshawa, ON, Canada), and a group of surgeons and surgical trainees located at the Centre Hospitalier de l'Université de Montréal (CHUM; Montreal). The BDA simulator was produced at maxSIMhealth and evaluated at the CHUM by surgeons, surgical residents, and medical students to assess its fidelity, functionality, and teaching quality.

The digital file that was developed for this project is shared publicly here: https://github.com/maxSIMhealth/Bile_Duct_Anastomosis_Mold. The use of all digital assets is bound by the terms and conditions of a Creative Commons Attribution-NonCommercial-ShareAlike 4.0 International Public License (CC BY-NC-SA 4.0). Subject to the terms and conditions of this public license, the authors and creators hereby grant a worldwide, royalty-free, non-sublicensable, nonexclusive, irrevocable license to reproduce and share the licensed material, in whole or in part, for strictly noncommercial purposes and produce, reproduce, and share adapted materials for noncommercial purposes only, under the same license. Please use this technical report as an acknowledgment for any research and description of educational activities that utilize any of these materials.

Furthermore, the materials included in this technical report are the intellectual property of many individuals (e.g., students, clinicians, engineers, and researchers). The creator (A. Dubrowski) and his institution (Ontario Tech University) do not warrant that these materials are complete, true, accurate, or non-misleading. By using these materials, the user agrees that the exclusions and limitations of liability set out in this disclaimer are reasonable. If the user does not think they are reasonable, they must not use these materials.

Inputs

Designers at maxSIMhealth developed the BDA simulator based on the guidance of two surgical oncologists and two general surgery residents (junior and senior residents, respectively, who both had prior BDA experience on in vivo models and patients) from the University of Montreal. The BDA simulator was produced using AM techniques such as 3D printing and silicone work. The following software was used to design the BDA simulator: Fusion360™ (Autodesk Inc., San Rafael, CA, USA) and Ultimaker Cura 3D printing software (Ultimaker B.V., Utrecht, The Netherlands). The following materials were used to construct the BDA simulator: Ecotough™ polylactic acid (PLA) filament material (Mississauga, ON), Ecoflex™ 00-20 FAST silicone (Smooth-On, Macungie, PA, USA), Ease Release 200 (Sculpture Supply Canada, Toronto, ON), Silc-Pig™ coloring (Smooth-On), and power mesh (80% nylon/20% spandex; Green Brook, NJ, USA, www.fabricland.com).

Process

Development of the BDA Simulator

**Figure 1:**
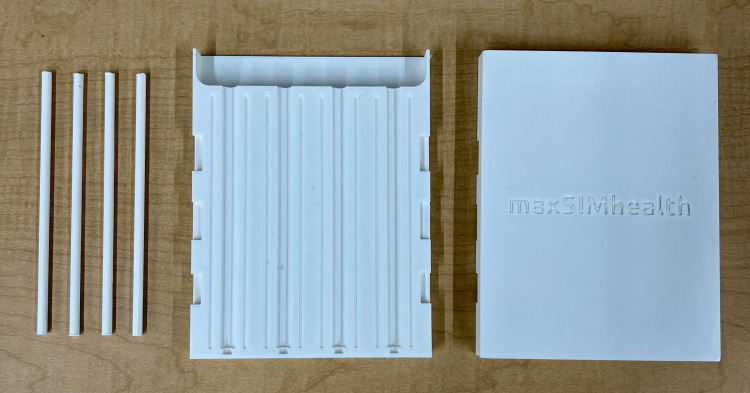
3D-printed mold for the BDA simulator. 3D, three-dimensional; BDA, bile duct anastomosis

The BDA simulator consists of a 3D-printed mold where a cut-out of power mesh can fit inside and silicone can be poured into. The mold for the BDA simulator shown in Figure 1 was designed in Fusion360™ to produce four bile ducts that were 140 mm long, had a 7-10 mm diameter, and a wall thickness of 1-1.5 mm. The design consisted of two halves that joined together to create four tunnels for the silicone to be poured into. Four rods were designed to fit within these four tunnels to ensure that the bile ducts were hollow. The design also includes small spaces between the four tunnels to reduce silicone leakage from the mold. Indents were designed on the outer edges of the mold to allow for easy pliability to remove the silicone from the mold. Finally, the top components of the outer halves of the mold were designed to create a funnel to allow for easy pouring of the silicone. The 3D design was sliced in Ultimaker Cura and 3D printed using Ecotough™ PLA material on an Ultimaker S5 3D printer (Ultimaker B.V.). 

**Figure 2:**
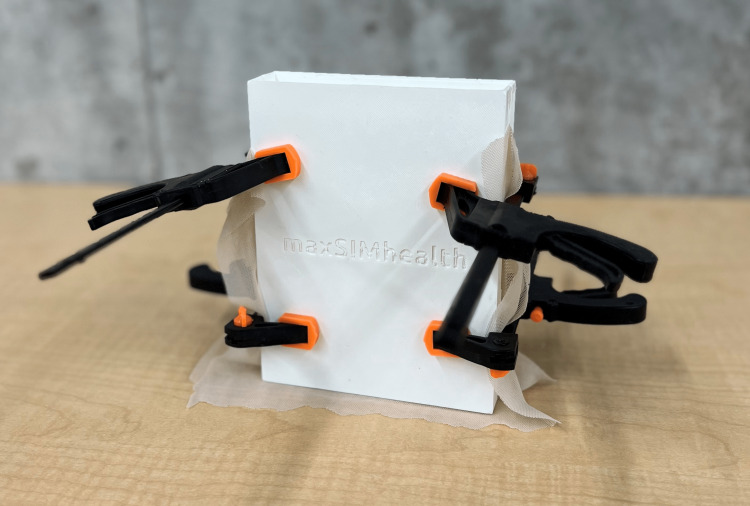
Assembled 3D-printed mold for the BDA simulator. 3D, three-dimensional; BDA, bile duct anastomosis

To produce the BDA simulator, first, the mold had to be assembled correctly so that the power mesh could be included before the silicone was poured into it. The power mesh was necessary for increasing the durability of the bile duct. First, all 3D-printed components were sprayed with Ease Release 200, a release agent, to allow for easy silicone removal and set to dry for 15 minutes. Once dried, one outer half of the mold was placed down on a stable surface facing up, then a rectangle of power mesh measuring 14 cm × 18 cm was placed on top so that the power mesh was lined up with the top part where the bile ducts would start, just at the bottom of the funnel. The four rods were lined up and placed within the tunnels of the outer half of the mold, on top of the power mesh. Another 14 cm × 18 cm layer of power mesh was added to cover the rods. To close the mold, the other outer half of the mold was lined up and placed on top to secure the two layers of the power mesh and the rods. To ensure that the mold would remain closed, four clamps (two at the top and two at the bottom) were secured onto the mold to remain closed in a vertical position. The assembled mold is shown in Figure 2.

**Figure 3:**
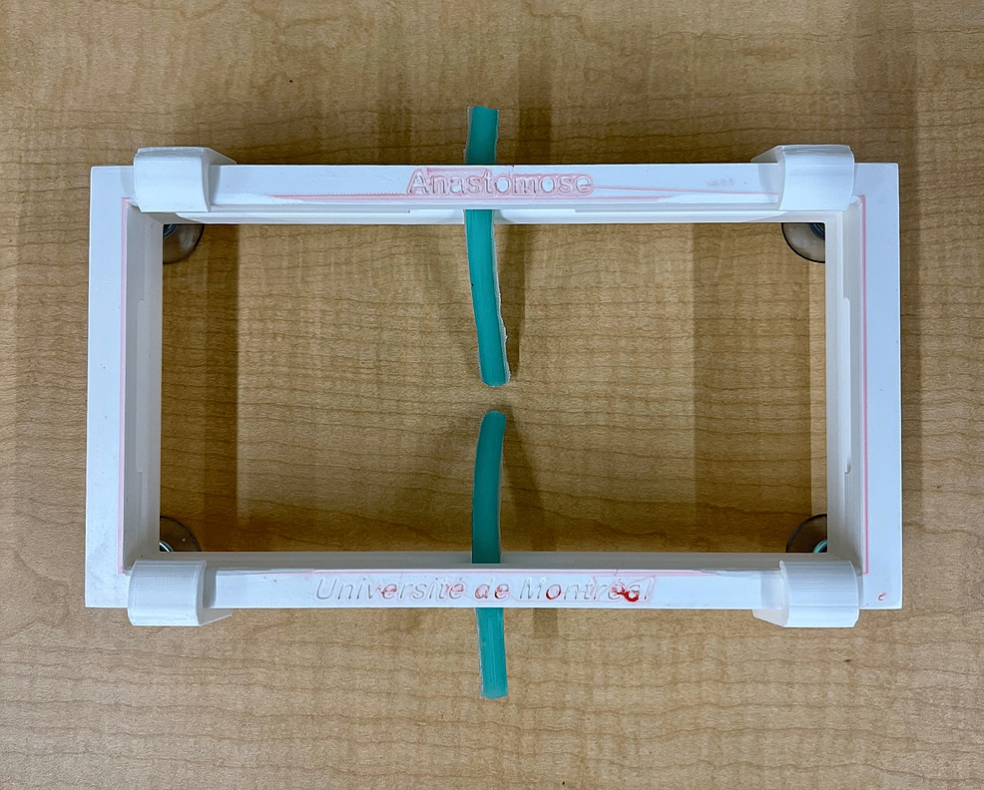
Complete BDA simulator with silicone bile ducts and 3D-printed maxSIMclamp. 3D, three-dimensional;  BDA, bile duct anastomosis

Once the mold and power mesh were assembled, the next step was to prepare the silicone. To fill the entire mold to make four bile ducts, 40 g of Ecoflex™ 00-20 FAST silicone was combined with green Silc-Pig™ silicone pigments to replicate the physical appearance of a bile duct. To reduce the number of air bubbles, the combined mixture of silicone was slowly poured into the funnel component of the mold. Once all the silicone was poured, the mold was tapped on the sides and lightly pounded onto the table to help the silicone move down the tunnels and release air bubbles. Additionally, toothpicks were secured at the top of the mold into the tunnels, within the top 1-2 mm of the bile ducts, to center the rod within the tunnels. The silicone was left to cure for 60 minutes and then removed from the mold. The removal involved removing the toothpicks and then separating the two outer halves of the mold. The rods were pushed out of the silicone, and the excess silicone and mesh were cut to produce four bile ducts. These silicone bile ducts were cut in half and secured within a 3D-printed clamp, called maxSIMclamp, which is described in a previous report [2]. This allows for each half of the bile duct to be secured so that it can be easily sutured by users. The complete BDA simulator is shown in Figure 3.

Assessment of the BDA Simulator

A total of nine informants tested and evaluated the BDA simulator at the CHUM. This testing group was from the CHUM and composed of six hepatobiliary surgeons, two surgical residents, and one medical student. A survey following a modified version of the Michigan Standard Simulation Experience Scale (MiSSES) template was used to assess the fidelity, functionality, and teaching quality of the BDA simulator [8]. There were four sections to the survey: (1) demographics, (2) self-efficacy, (3) fidelity, and (4) overall rating. In total, there were 21 questions, as shown in Table 1. The survey was created in Google Forms and was printed to have physical copies for the nine users to fill in.

**Table 1:** Survey questions for the BDA simulator. BDA, bile duct anastomosis

Question no.	Questions
	Demographics
1	What is your specialty?
2	If you are a student, what year of your program are you in?
3	If you are a practicing physician, how long have you been practicing for?
	Self-efficacy
4	This model helped improve my knowledge of the procedure in scope.
5	This model helped improve my confidence in performing the procedure in scope.
6	This model helped improve my ability in performing the procedure in scope.
7	Share comments/suggestions regarding the model that may improve self-efficacy.
	Fidelity
8	This bile duct simulator used has anatomically accurate characteristics/features.
9	On a scale of 1 to 5, how accurate did the bile duct feel?
10	On a scale of 1 to 5, how well were you able to suture the bile duct?
11	On a scale of 1 to 5, how durable would you say the bile duct simulator is?
12	Comments/suggestions to improve the fidelity of the bile duct simulator.
13	Share comments/suggestions to improve the functionality of the bile duct simulator.
14	On a scale of 1-5, how difficult was it to use the model?
15	Share comments/suggestions to make the simulator less difficult to use.
	Overall rating
16	Overall, the bile duct simulator was a helpful training tool for the procedure in scope.
17	For the evaluation of the model...
18	Share comments/suggestions to improve the bile duct simulator overall.
19	Other than the simulator used today, have you used a bile duct simulator in the past?
20	If another bile duct simulator has been used in the past, what was the simulator called, and/or can you provide a quick description of the simulator?
21	If you have used another bile duct simulator in the past, how does that one compare to the simulator used today?

Outcome

Development of the BDA Simulator

The total cost of the bile duct simulator is broken down in Table 2, which includes the maxSIMclamp described in a previous report [2], the bile duct mold, and four individual silicone bile ducts. The total cost to produce the BDA simulator with four bile ducts is Canadian dollar (CAD) 23.41, including taxes.

**Table 2:** Cost breakdown of the BDA simulator. BDA, bile duct anastomosis; CAD, Canadian dollar; PLA, polylactic acid

Simulator component	Material	Amount used	Cost (in CAD with taxes)
BDA mold	PLA	227 g	12.30
maxSIMclamp	PLA	160 g	8.67
Power mesh	80% nylon, 20% spandex	252 cm²	0.21
Four bile ducts	Ecoflex™ 00-20 FAST silicone	40 g	2.23
		Total cost	23.41

Assessment of the BDA Simulator

The survey data were considered ordinal data [9,10] and are presented using descriptive statistics, both as frequencies of distribution as per each question and mean and standard deviations (SDs). This was chosen as our informant numbers were low and the point of the analysis was to inform the design rather than to provide evidence of validity. The results from the survey are broken down into quantitative data (Table 3) and qualitative data (Table 4). 

**Table 3:** Quantitative survey data for the BDA simulator. *It requires extensive improvements before it can be considered for use in training. **It requires minor adjustments before it can be considered for use in training. ***It can be used in training but should be improved slightly. ****It can be used in training with no improvements. BDA, bile duct anastomosis

Question no.	Scale 1 (strongly disagree) to 5 (strongly agree)					Total	Average response	Standard deviation
	1	2	3	4	5			
4	0	0	2	1	0	3	3.33	0.58
5	0	0	2	1	0	3	3.33	0.58
6	0	0	1	0	0	1	2.00	N/A
8	0	2	2	3	2	9	3.56	1.13
	Scale 1 (not accurate) to 5 (very accurate)							
	1	2	3	4	5			
9	0	3	2	4	0	9	3.11	0.93
	Scale 1 (not well) to 5 (very well)							
	1	2	3	4	5			
10	0	1	0	0	8	9	4.67	1.00
	Scale 1 (not durable) to 5 (very durable)							
	1	2	3	4	5			
11	0	0	1	3	4	8	4.38	0.74
	Scale 1 (very difficult) to 5 (not difficult)							
	1	2	3	4	5			
14	0	0	0	1	8	9	4.89	0.33
	Scale 1 (strongly disagree) to 5 (strongly agree)							
	1	2	3	4	5			
16	0	0	0	4	4	8	4.50	0.53
	Option 1^*^	Option 2^**^	Option 3^***^	Option 4^****^				
17	0	3	2	3		8	3.00	0.93

**Table 4:** Qualitative survey data for the BDA simulator. BDA, bile duct anastomosis

Question no.	Comments
7	“Very easy to use, even if I never sutured a bile duct in real life. Less fragile than I would've thought. Allows me to relatively strongly tighten the knot without worrying about ripping the tissue.”
12	“Make it thinner Make color beige Look thinner A little too soft (in terms of texture). Seems more resistant than real bile ducts, however real bile ducts can tear under pressure (a badly controlled suture for example) Be careful of the instrument used, you need the appropriate suture, needle and needle driver.”
13	“Too resistant, a real bile duct would in practice rip more easily.”
18	“Needs a little more ‘fibrousness’. This feels too soft.”
20	“3D-printed bile duct also.”
21	“It was more rigid, and probably less realistic than this one This one is better, great job!”

The results for the quantitative data (Table 3) indicate that some questions were skipped by many of the informants; therefore, we will only comment on the results where at least eight of nine informants responded. The fidelity component of the BDA simulator appeared to be its weakest point in terms of the overall anatomical accuracy of the characteristics of the BDA simulator rated a 3.56/5 (SD = 1.13). More specifically, the accuracy of the feel of the bile duct has been rated at an average of 3.11/5 (SD = 0.93). However, when users compared this BDA simulator with other 3D-printed simulators they have used in the past, they indicated that this one is more realistic. The functionality of the simulator seemed to be a highlight, with the average response of how well the users were able to suture the BDA simulator being 4.67/5 (SD = 1.00), while the durability was rated 4.38/5 (SD = 0.74). Looking at the general comments regarding the fidelity and functionality of the BDA simulator in Table 4, many suggestions to improve the fidelity were to make the bile ducts thinner and for it to be recolored beige. While the BDA simulator was rated highly for its durability in Table 3, the comments from Table 4 suggest that the durability of the simulator makes it less accurate and should be made softer. Another noteworthy result is the teaching quality of the BDA simulator, which was rated 4.5/5 (SD = 0.53) for its use as a training tool. Finally, when asking whether this tool is acceptable to be used for training, the average response was option 3 (SD = 0.93), indicating that it can be used in training but should be improved slightly.

## Discussion

First, this report aimed to describe the development of a BDA simulator using AM techniques such as 3D printing and silicone work. This was done collaboratively by taking the information provided by surgical experts to design and produce a BDA simulator that met users’ needs. As a result, a functional and cost-effective BDA simulator was produced for CAD 23.41 and will only cost CAD 2.23 to replace the silicone bile ducts if needed. The BDA simulator was designed and produced following a design-to-value approach, meaning cost was not taken into consideration throughout the process so that the needs of users are fully met [11]. Despite this, the results indicate that this simulator is cost-effective in comparison to other simulators on the market. Specifically, the pancreaticojejunal anastomosis model from LifeLike BioTissue is CAD 87.00 before tax [12]. Because of the cost-effective nature of the simulator, and its overall acceptability as a training tool for general surgery residents, this BDA simulator is ideal for use in an SBME context. Due to its affordability, generally the main inhibitor of training in those areas, rural and remote areas would greatly benefit from this simulator [13].

Additionally, the cost-effective nature of the BDA simulator makes it an excellent candidate for decentralized education, which can play a major role within the Internet of Things (IoT) concept used in resident surgeon training [2,14]. While the IoT concept in surgery largely focuses on telesurgery and telemedicine, it can also be applied to surgical education. This idea of using technology for surgical training has been tested when a handsewn bowel anastomosis simulator was developed and used to train surgical residents via an online learning management system called the gamification educational network (GEN) [2]. GEN provided learners with the ability to learn this surgical skill online from their homes while using the developed simulator, with the results indicating that the surgical residents liked this training platform and found that it helped improve their understanding of the technique [2]. Therefore, using GEN alongside the BDA simulator can fit within the IoT paradigm to advance surgical education.

Second, this report aimed to provide an initial evaluation of the BDA simulator. A group of nine surgeons and surgical residents indicated in the initial evaluation that the simulator needed to improve on its fidelity by being thinner, softer, and a different color. More optimistically, it was said to be more realistic compared to previous simulators and was ranked highly for being accepted as a training tool for general surgery residents. While it is an advantage that of our nine informants, six are hepatobiliary surgeons who have a lot of experience in performing this procedure on a human, however, this resulted in the questions regarding self-efficacy being disregarded by that population. Therefore, it is a limitation that we only had the perspective of three surgical residents and medical students, so we could not accurately assess the perceived self-efficacy when using the BDA simulator from a learner's point of view. However, as the focus of this report was on the initial evaluation of the BDA simulator, its effectiveness as a learning tool can be assessed in the future.

## Conclusions

This report described the development of a BDA simulator collaboratively through the use of a design team and a group of surgeons and surgical residents using AM techniques. The BDA simulator then went through an initial evaluation to assess the fidelity and teaching quality of the BDA simulator. The results indicate that a cost-effective and anatomically accurate BDA simulator can be designed and manufactured using AM techniques such as 3D printing and silicone work. With a few small adjustments, such as making the bile ducts thinner, softer, and more realistically colored, this BDA simulator can become an excellent tool for training general surgery residents.
